# Quantity of Food

**Published:** 1896-09

**Authors:** 


### Quantity of Food.

To the question which has been frequently asked, What quantity of food is best adapted for preservation of health? no satisfactory answer can be given, without a reference to the habits, occupation, and age of each individual, the degree of health he enjoys, as well as the season of the year and other circumstances. As a general rule, it will be found that those who exercise in the open air, or follow laborious occupations, will demand a larger amount of food than the indolent or the sedentary. Young persons, also, require more than those in advanced years, and the inhabitants of cold more than those of warm climates. We say this is a general rule, for very many exceptions are to be found in each of the particulars. Thus, we not infrequently find that one individual requires more food to support his system than another of the same frame of body and trade, and who partakes of the same degree of exercise. In fact, one person will support his strength, or even become robust, upon the same quantity of food, which will occasion in another debility and emaciation.

If we refer to the brute creation, which is guided in this respect by an instinct which but rarely errs, we find that one horse requires more food than another of similar age and size, and with the same degree of exercise; and if his accustomed quantity be diminished he will become thin and spiritless. This is true, also, in respect to other animals.

Every person arrived at the age of maturity, or even before, should be able to judge for himself as to the quantity of food proper for each meal, as well as to the frequency with which it should be repeated during the day. Few appear, however, to be aware of the important fact that the body is nourished, not in proportion to the amount, or even the nutritious qualities of the food which is consumed, but to the quality which the stomach actually digests. All beyond this disorders the stomach; and if the excess be frequently indulged in, the latter becomes finally incapable of converting into nutriment even a sufficiency for a support of the system. Most persons act as though the strength, vigor, and health of the body rise in proportion to the load of food they are capable of forcing daily in the stomach; and hence, overfeeding is the common error, at least in our own country. A slight deficiency of food is, however, far less injurious than too great an amount. The old maxim, "If health is your object, rise from the table before your appetite is sated," is founded in truth, and though the epicure will sneer at it, yet were he wisely to adhere to it he would save himself from many a gloomy hour of pain and suffering.

When the stomach is not laboring under disease, and the individual is otherwise in health, the natural appetite is one of the very best guides—the only one, indeed, as to the time for eating, as well as to the quantity of food. Whenever such appetite exists, wholesome food may and ought to be taken; we should cease from eating the moment it is satisfied.

The eccentric author of Emillius makes the following very judicious remarks in reference to the diet of children:

"Whatever regimen you prescribe for children, provided you only accustom them to plain and simple food, you may let them eat, run, and play as much as they please, and you may be sure that they will never eat too much, or be troubled with indigestion. But if you starve them half the day, and if they find means to escape your observation, they will make themselves amends, and eat till they are sick.

"Our appetite is only unreasonable; because we choose to regulate it by other laws than those of nature. Always laying down arbitrary rules, governing, prescribing, adding, retrenching, we never do anything without the scales in our hands; and this balance is formed according to the measure of our fancies and not according to that of our stomachs."

The foregoing remark will equally apply to the adult as well as to the child. It is important, however, that "the balance" of the stomach be not rendered untrue by the acts of cookery—in other words, that an artificial appetite be not created by a variety of luxurious dishes—by sauces, condiments and wine.

It is surprising how often the stomach, within a very short space of time, may be artificially excited to a renewed desire for food. The man, however, who eats under such circumstances, must not be surprised at his uncomfortable feelings and frequent ailments. He has scarcely more right to expect health and long life than the individual who would attempt to nourish himself with poison.—*Dietetic and Hygienic Gazette.*

